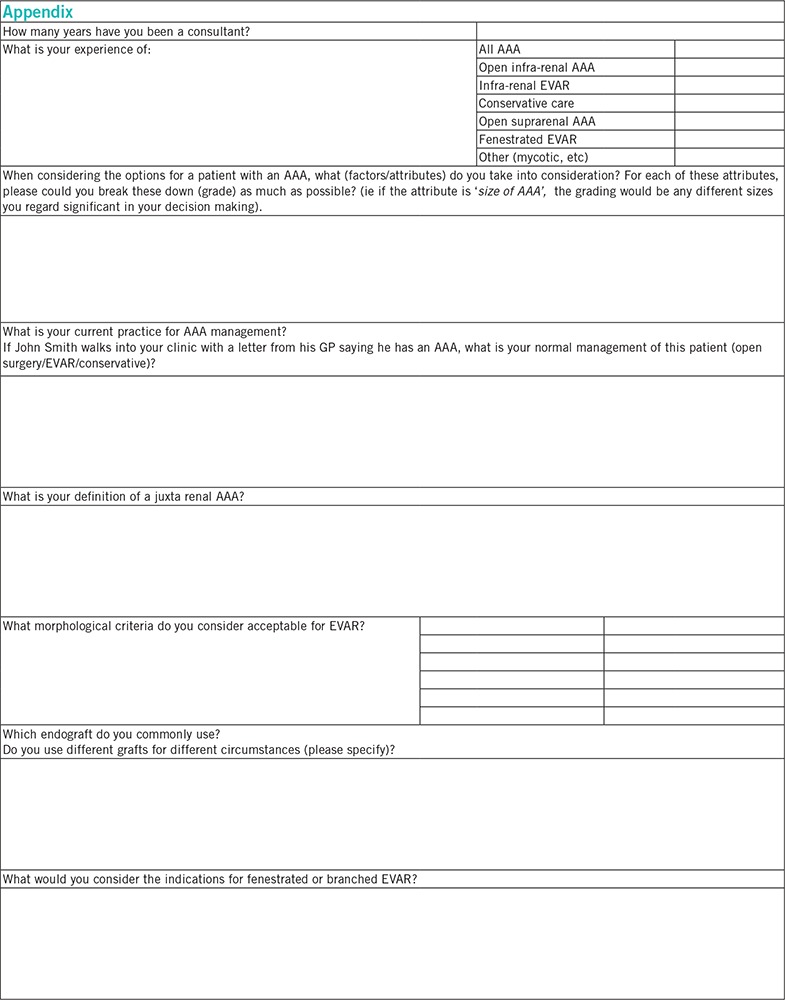# Current practice of aneurysm management in the UK

**DOI:** 10.1308/003588414X13824511650416

**Published:** 2014-01

**Authors:** J Cross, T Richards

**Affiliations:** University College Hospital, London,UK

**Keywords:** Aortic aneurysm, Supra-renal aneurysm, Juxta-renal aneurysm, EVAR

## Abstract

**INTRODUCTION:**

The aim of this study is to establish the current practice of aneurysm management, to assess the introduction of fenestrated endovascular aneurysm repair (FEVAR) and to establish the criteria for its use and its role in the UK.

**METHODS:**

All UK centres performing FEVAR and centres with an established interest in infra-renal endovascular aneurysm repair (EVAR) were invited to respond to an open-ended questionnaire about abdominal aortic aneurysm (AAA) management.

**RESULTS:**

A response was obtained from over 90% of UK FEVAR centres. Results showed marked regional differences in aneurysm management, in particular with regard to indications for complex aneurysm management.

**CONCLUSION:**

The trend in the UK is towards endovascular repair. However, there are still variations in unit policies, indicating regional differences in patient management.

The process of the introduction of a new surgical technique is much debated. Traditional methods include presentations at surgical meetings, publication of case reports and case series, and the development of large, observational and often retrospective studies. As modern surgery has developed, new innovations may be refinements of previous techniques, and these often confer smaller and less striking improvements than those previously reported.

Randomised controlled trials (RCT) are commonly described as the ‘gold standard’ of surgical research, and are designed to clearly show any benefit imparted by an intervention. However, good quality trials usually take time to set up, require a large number of patients, are expensive, and the results may not be immediately apparent. Technology may evolve too fast for RCTs to be useful, and at times they are not possible or appropriate to perform. The management of complex aneurysms is one example where technology has currently exceeded good quality clinical research evidence.

EVAR has revolutionised the management of aneurysm disease. There is Level 1 evidence establishing it as a viable alternative to open repair^1^ for infra-renal aneurysms. Its use has increased rapidly, and the *National Vascular Database Audit 2009*^2^ showed that over 44% of AAAs in the UK underwent EVAR. Although it was first described by Parodi in 1991,^3^ guidelines establishing EVAR criteria were not published until 2003^4^ and have only recently been updated^5^ to incorporate later generation graft design.

The development of fenestrated and branched endografts has enabled endovascular intervention for juxta-renal/thoraco-abdominal aneurysms. However, benefits of FEVAR over open repair may be less than those seen with standard infra-renal EVAR, and questions on the validity of FEVAR for juxta-renal aneurysms have arisen. The heterogeneity between the case series and the lack of high-quality evidence have made the indications and the role of FEVAR unclear. Consequently, in the UK, FEVAR is not universally available. The aim of this study is to establish the current practice of aneurysm management, to assess the introduction of FEVAR, and to establish the criteria for its use and its role in the UK.

## Methods

A full list of UK FEVAR centres was obtained from the Cook database. The British Society of Endovascular Therapy ensured the representation of at least one individual from each UK vascular centre specialising in complex aneurysm repair. All UK centres performing FEVAR and centres with an established interest in infra-renal EVAR were invited to participate.

A questionnaire was developed regarding current practice of AAA management (see Appendix 1). The questionnaire was undertaken either as a telephone interview or via email. Both consultant vascular surgeons and consultant interventional radiologists were invited to participate.

1. The questions were broadly divided into four sections:
2. Previous aneurysm experience.
3. Current practice of aneurysm management.
4. Definition of juxta-renal aneurysm.
5. Indications for FEVAR.

The questions were specifically open-ended and worded to avoid prompting answers.

## Results

### Previous aneurysm experience

Of the 45 UK consultants invited to participate, 26 responded. Of these, 4 were primarily consultant radiologists and 22 were primarily vascular surgeons. A response was obtained from over 90% of UK FEVAR centres.

**Figure 1:**
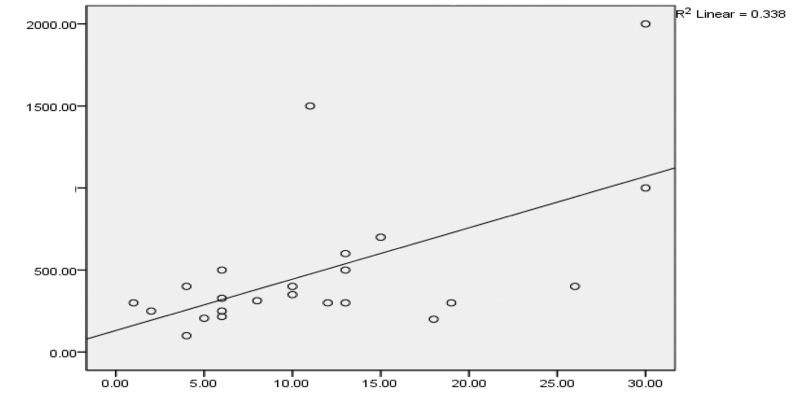
Years of consultant experience against number of aneurysms managed

The median number of years in consultant position was 10.5 (range 1–30). The median number of aneurysms managed was 328 (range 100–2,000) (see Fig 1). The majority of surgeons had managed fewer than 500 aneurysms. There was a significant correlation between years of experience and the number of aneurysms managed (Pearson correlation coefficient 0.581, *p*=0.004).

**Figure 2:**
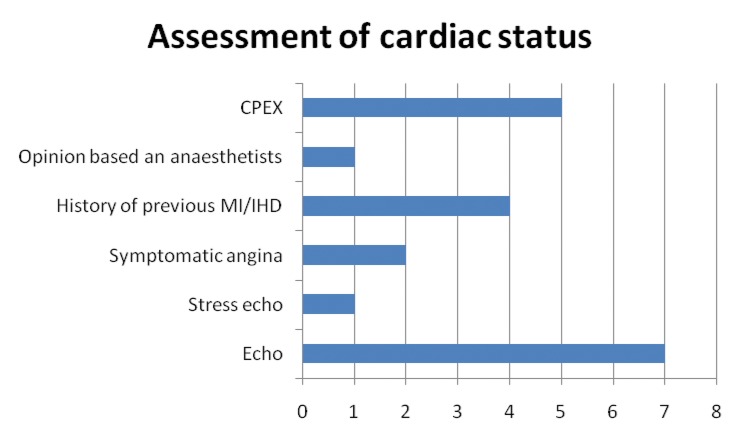
Assessment of cardiac status

The median number of open infra-renal aneurysms managed was 125 (range 20–400) and the median number of infra-renal EVAR managed was 160 (range 8–800). Most consultants had limited experience of fenestrated EVAR: over half (54%, *n*=14) had been involved with 10 or fewer, and only 23% (*n*=6) had been involved with 50 or more. The median number of open supra-renal aneurysms managed was 14 (range 1–400).

### Standard practice

Of the consultants, 10/26 stated that EVAR was their first choice. Almost all consultants said they would discuss both options with the patient and that they would generally recommend a modality based on patient fitness. Only three consultants specifically said that the management decisions were made after a multi-disciplinary team discussion. Four consultants said they used scoring systems to help predict patient outcomes.

### Patient factors

All the consultants cited fitness for surgery when deciding management options for aneurysms, but the definition of this varied considerably.

There was no concurrence on assessment of cardiac status. A cardiac echo was the most commonly cited investigation; however, a significant ejection fraction was not universally agreed. Although respiratory disease was cited by 9 consultants and renal disease by 11, the cut-off levels for significant parameters varied considerably. Seven consultants cited exercise tolerance, and it was generally agreed that patients should be able to climb one flight of stairs. Other factors taken into consideration were the ability to self-care, life expectancy, obesity, symptomatic aneurysms, claudication, scoring systems and the presence of a hostile abdomen.

### Aneurysm size

It was agreed that 5.5cm is the cut-off for a fit male, but two consultants said they would consider repair in females at 5cm. Most consultants said they would wait until the aneurysm had reached 6–7cm before intervening on an unfit patient.

### Age

Four consultants said age alone was not considered when deciding aneurysm management. Of those that mentioned age, it was agreed that open repair was preferable for patients under the age of 60, and EVAR was preferable for patients over the age of 80. However opinions varied as to the best practice for the 60–80 age group.

### EVAR morphology

The definition of an acceptable neck length for standard infra-renal EVAR varied considerably. Although it was agreed that a straight, thrombus-free neck of >15mm was suitable, 12 consultants said they would accept a neck length of 10mm, and three said they would accept a neck length of up to 8mm. Only five consultants said they would not accept angulation of more than 60 degrees, and four consultants said they would accept neck angulation of up to 90 degrees. Where cited, it was generally agreed that the iliacs should be greater than 6mm and not heavily calcified.

### Graft type

The most commonly used graft was the cook zenith (*n*=17 consultants used this as a first line graft) followed by the medtronic endurant (*n*=10). The Aorfix was commonly used for angulated necks.

### Juxtarenal definition

The definition of a juxtarenal aneurysm was varied. Fourteen consultants defined it as being the need to clamp above one or more renal arteries; two defined it as a Crawford type 4 aneurysm; 3 only as ‘short neck’; 6 defined it as a neck length of less than 10mm; two defined it as a neck length of <5mm; and one as less than 3mm. One consultant defined it as ‘seal zone above the renal arteries’.

### Indications for FEVAR

There was little agreement on the indications for FEVAR. Two consultants were unsure when to use FEVAR; 10 felt FEVAR should be used for patients who were unfit for open repair if standard EVAR was not suitable; ‘complex aneurysm’/tho-racoabdominal aneurysm/juxtarenal aneurysm was cited by 15 consultants; and short/hostile neck was used by 10 consultants. However, only four consultants gave a neck length: three stated less than 10mm and one less than 15mm.

## Discussion

This audit presents a snapshot of the wide variation in aneurysm management in the UK. Of particular interest is the poor concurrence on the indications for FEVAR and the definition of a juxta-renal aneurysm.

Although the questionnaire was deliberately open-ended, this may have limited the responses and led to a misunderstanding about the expected answers. The respondents may have elaborated more if prompts had been given, and they may have omitted some answers owing to the nature of the questions. The questionnaires conducted over the telephone were less likely to have been misunderstood.

The definition of fitness for surgery and the standard preoperative investigations varied between institutions. While severe cardiac disease is a contra-indication, the methods of definition and the cut-off level at which it was deemed too unsafe were not universal. This was also the case for respiratory and renal disease. Exercise tolerance and the ability to climb stairs, although very subjective, are commonly used parameters. However, the EVAR II^6^ trial showed clinical judgement to be a satisfactory method of deciding patient fitness. There was agreement that an unfit patient should wait until the aneurysm had reached >6cm, regardless of intervention modality. Although EVAR II showed a lower than anticipated rupture rate, the all-cause mortality in this group was high and the benefit of EVAR in someone with a limited life expectancy is not clear.

It was agreed that an aneurysm size of 5.5cm was the point to intervene in a fit patient, regardless of treatment modality. This is based on the results from the UKSAT.^7^ The results from CESAR^8^ and PIVOTAL^9^ have not shown any benefit for EVAR with early intervention.

Patient age is a controversial area and opinion varied, particularly in the management of patients aged 60–80 years. Although it is generally agreed that EVAR is the better option for older patients, there has been some reluctance to insert grafts into young patients. This is based on the high reintervention rates associated with EVAR and the radiation dose from repeated surveillance CT scans. Surveillance duplex is becoming the modality of choice and, as graft design is improving, reintervention rates are decreasing.

Acceptable aneurysm morphology suitable for a standard infra-renal graft varied considerably, and over half the consultants would be prepared to insert a standard infra-renal graft outside the manufacturers’ instructions for use. Some consultants would not consider a standard EVAR in a neck of less than 15mm, while others would be prepared to accept a neck length of up to 8mm. Although graft design has improved considerably and grafts are now licensed for up to a 10mm neck, previous studies have shown that short and/or angulated necks (10–11mm) are associated with a higher endoleak and a subsequent re-intervention rate.

This also showed a large overlap with the indications for FEVAR. Although FEVAR is often considered for aneurysms requiring infra-renal grafts used outside the manufacturers’ instructions, our survey showed that, in a significant number of cases, standard grafts are used where other centres would consider a fenestrated graft to be most suitable. It is interesting to note that FEVAR use was often considered for unfit patients. Evidence for the benefit for unfit patients is poor and, given the EVAR II data, it is unclear whether FEVAR should be considered in unfit patients at all.

There was no clear definition of a juxta-renal aneurysm. The old definition relating to clamp placement is now obsolete in the endovascular era, and a new common definition should be agreed to enable uniformity in management and the ability to accurately compare morphology. This is particularly apparent in the use of FEVAR.

This survey has highlighted that the indications for use and the role of FEVAR are unclear. Only a small number of UK consultants have significant experience of FEVAR and, although its use is increasing, its introduction remains haphazard with no guidelines. Differences in opinion usually indicate a lack of good-quality evidence and the need for further research.

It appears that the trend in the UK is heading towards endovascular repair, and 10/26 consultants stated that this was their first choice. However, there are still variations in unit policies, indicating regional differences in patient management. This study has highlighted the need for further guidelines regarding the role of FEVAR.